# Renal markers for assessment of renal tubular and glomerular dysfunction

**Published:** 2013-07-01

**Authors:** Dejan Spasovski

**Affiliations:** Department of Rheumatology, University Clinical Centre, Skopje, Republic of Macedonia

**Keywords:** Proximal tubule, Enzyme, Kidney

**Implication for health policy/practice/research/medical education:**

There are approximately 40 different enzymes in the urine with different origin. They originate from the kidneys, urinary tract epithelium and urinary tract glands, plasma and blood cells. Increased enzymatic activity can be a reflection of disease activity and of the residual functional capacity of the kidney.

There are approximately 40 different enzymes in the urine with different origin. They originate from the kidneys, urinary tract epithelium and urinary tract glands, plasma and blood cells (1). Subcellular locations of these enzymes are:

1. Membranous (Alanine Amino Peptidase; AAP, γ-glutamyl transferase; γ-GT)
2. Lysosomal (N-acetyl-β-(D)-glucosaminidase activity; NAG)
3. Mitochondrial (Malate dehydrogenase: MDH)
4. Cytoplasmic (LDH)

However, proximal tubules of the kidneys have a dominant role in their excretion. Examination of the brush border epithelium (BBE) of the proximal tubules confirms that alanine amino peptidase (AAP ) (90%), alkaline phosphatase, ALP (70%) and γ-glutamyl transferase, γ-GT (50%), constitute the largest part of the total activity of these enzymes in the kidney (1). Because BBE is very sensitive to insults, these and other enzymes can be used as markers for secondary renal damage in the setting of different diseases, medicines and toxins (2). Increased enzymatic activity can be a reflection of disease activity and of the residual functional capacity of the kidney.

Elevation of the urinary enzymes may indicate renal tubular damage. Urinary enzymes such as microsomal AAP and γ-GT can be used to detect early acute renal tubular damage which may be provoked by immunosuppressive medications, contrast media, antibiotics and chronic inflammatory disorders such as rheumatoid arthritis. Renal tubular damage could be a visceral manifestation of systemic diseases too (3).

The standard routine parameters which are used for assessment of glomerular filtration rate (GFR); have a relatively low sensitivity due to the large functional renal reserve (4). Up to 50% of renal functional capacity would be lost before any increase in blood urea nitrogen and appearance of proteinuria. Renal function and integrity can be determined by many methods such as immune, radiologic, cytological analyses, but an important modality is biochemical analyses, as non-invasive methods which have a major role in the early detection of some pathological conditions. The regulation of activity of enzymes and their isoenzymes in urine is very important because their activity in serum has small diagnostic value.

Pathogenic mechanisms leading to the destruction of epithelial cells of proximal tubules that are responsible for the appearance of enzymuria are; immune mechanism, complement, lysosomal enzymes and tubular obstruction by cell debris, protein cylinders, toxic noxes, medicaments or proteinuria. Each of them, to a different degree, in a direct or indirect way, contributes to the release of biochemical markers in urine.

## Renal markers for assessment of renal dysfunction

Some classes of measured proteins in urine are used for assessment of asymptomatic renal dysfunction as following:

1. Enzymes with high molecular weight, which are not usually filtered in the glomerulus, with genesis in the proximal tubule (NAG)
2. Medium-sized proteins which are normally filtered in the glomerulus in a very small quantity, while the biggest part is resorbed in the tubules (microalbumin) (5-10)

Of all urine enzymes, the most studied protein is U-NAG. It is an enzyme that belongs to the hydrolase class usually presents in the lysosomes of proximal tubular cells (11). In the human tissue and in biological liquids, two main forms of enzyme exist: A (Acid) and B (Basic) (12-14). The percentage of A isoform (U-NAG-A) is the dominant form in normal urine (15,16). At the end of cell maturation process, it is placed in the resolved form of cytosol. Its excretion is related to the exfoliative turnover and is signed as functional enzymuria. B isoform (U-NAG-B) is dependent on maturation and is more closely connected with the basal membrane where it appears. Because of this localization of B isoform, large amount of NAG is released in tubular lumen only in cases of cytolytic tubular lesion. Its presence in the urine is in related to the cell lysis and, because of that, is signed as lesion enzymuria (17,18). NAG can be detected in circulation but plasma NAG cannot pass through an intact glomerular membrane due to its large molecular weight (140000 daltons). In healthy people, urine NAG is a representative of the total amount which is released from the walls of renal tubular cells (16) and is a very sensitive marker for renal tubular damage (19-21).

Albumin (molecular weight from 66 KDa) is quantitatively the most important protein in plasma and urine. Approximately, it constitutes up to 30% of proteins in the urine and it appears to be a good indicator for assessment of the change of glomerular permeability. The change in glomerular permeability occurs in diabetic and hypertensive nephropathy, nephrotic syndrome, pre-eclampsia and glomerulonephritis. Urine albumin excretion has a high individual variability and depends on physical activity or food. From the pathophysiological aspect, microalbuminuria can be caused by increased glomerular permeability to albumin, increased glomerular pressure and/or reduced tubular albumin reabsorption. Renal vascular endothelium is intimately involved in the regulation of these processes (22,23).

Alanine aminopeptidase (AAP) similar like leucine peptidase, hydrolyzes to peptides, amids and p-nitroanilide. During hydrolization of peptides N-terminal amino acid is separated. The activity of AAP is determinated by methods similar to those used for measurement of leucine aminopeptidase. In this method L-alanine-4-nitroanilide is used as a substrate. The catalytic concentration of AAP is directly proportional to the absorption of p-nitroanilide measured on 405 nm. (Reference rates: AAP in urine 0.25-0.75 U/mmol creatinine).

AAP is found in many tissues, such as kidneys, intestine, lung and liver. AAP in different organs has different electrophoretic conductivity. This enzyme has at least five different isoenzymes that could be separated from each other electrophoretically, with ion change chromatography or immunologically. In normal serum only one isoenzyme is found, while in hepatobilliary or pancreatic disease additional fractions are found. The enzyme is detected in urine.

## Author’s contribution

DS was the single author of the paper.

## Conflict of interests

The author declared no competing interests.

## Ethical considerations

Ethical issues (including plagiarism, data fabrication, double publication) have been completely observed by the author.

## Funding/Support

None.
